# High stress related to COVID-19 among health workers in the Plateau Central healthcare region (BURKINA FASO): a cross-sectional study

**DOI:** 10.3389/fpubh.2023.1162707

**Published:** 2023-06-05

**Authors:** Solo Traoré, Désiré Lucien Dahourou, Boyo Constant Paré, Yemboado Diedonné Lompo, Wendlassida Josiane Kaboré, Wind-La-Sida Abd-El-Aziz Ouédraogo, Datouo Thomas Kambou, Wenddinda Rabbaly Adeline Salou, Delphin Kaboré, Abdoulaye Ouédraogo, Siaka Sia, Mady Zorné, Boezemwendé Ouoba, Oumar Guira

**Affiliations:** ^1^Department of Medicine and Medical Specialties, Ziniaré Regional Hospital, Plateau Central Healthcare Region, Ziniaré, Burkina Faso; ^2^Department of Biomedical and Public Health, Research Institute of Health Sciences, Ouagadougou, Burkina Faso; ^3^Training and Research Unit in Health Sciences, Department of Public Health, Joseph Ki-Zerbo University, Ouagadougou, Burkina Faso; ^4^Gynecology and Obstetric Department of the Regional Hospital Center of Ziniaré, Plateau Central Healthcare Region, Ziniaré, Burkina Faso; ^5^Boussé Healthcare District, Plateau Central Healthcare Region, Boussé, Burkina Faso; ^6^Zorgho Healthcare District, Plateau Central Healthcare Region, Zorgho, Burkina Faso; ^7^Ziniaré Healthcare District, Plateau Central Healthcare Region, Ziniaré, Burkina Faso; ^8^Regional Healthcare Directorate of the Plateau Central Healthcare Region, Ziniaré, Burkina Faso; ^9^Head Directorate of the Regional Hospital Center of Ziniaré, Plateau Central Healthcare Region, Ziniaré, Burkina Faso; ^10^Training and Research Unit in Health Sciences, Joseph Ki-Zerbo University, Ouagadougou, Burkina Faso; ^11^Internal Medicine Department, Yalgado Ouédraogo University Hospital, Ouagadougou, Burkina Faso

**Keywords:** COVID-19, PSS-10, stress, health workers, Plateau Central healthcare region

## Abstract

**Background:**

The COVID-19 pandemic challenged the mental wellbeing of health workers. The objective of this study was to assess health workers' perceived stress during the response to COVID-19 in the Central Plateau region (Burkina Faso).

**Methods:**

We conducted a cross-sectional study of health workers in the Central Plateau health region from September 20 to October 20, 2021. Agents' perceived stress was assessed by the Perceived Stress Scale (PSS-10). Factors associated with high stress (PSS-10 score ≥ 27) were identified by logistic regression.

**Results:**

A total of 272 officers participated in the survey. The mean PSS-10 score was 29.3 points (standard deviation: 6.2). Three out of ten agents (68%) had a high level of stress. The main sources of stress were the risk of being exposed to contamination (70%) and being the source of contamination (78%). Working at the referral health center [adjusted odds ratio (aOR): 2.29; 95% confidence interval (95% CI): 1.19–4.41], the hospital as the main source of COVID-19 information (aOR: 1.17; 95% CI: 1.01-3.04), fear of COVID-19 patients being managed at one's center (aOR: 1.8; 95% CI: 1.06–3.07) were factors associated with high health worker stress levels during the first wave of COVID-19.

**Conclusion:**

The COVID-19 pandemic caused high stress among health care workers in Burkina Faso. Psychological support for health center workers in responding to future epidemics would improve their mental health.

## Background

The pandemic of SARS-CoV-2 (Severe Acute Respiratoy Syndrome Coronavirus 2) infection, responsible for COVID-19 disease, by its sudden, global and uncertain character, also proves to be very anxiety-provoking for the general public and makes it necessary to evaluate its consequences on mental health (1–8). As of August 7, 2022, 584,211,310 cases have been detected and 6,417,886 deaths recorded worldwide (9). In Burkina Faso, since the confirmation of the first case on March 09, 2020 in Ouagadougou, the spread of the disease was fearfull due to the highly contagious nature of the virus, the inadequacies of our health system and the intensity of socio-economic exchanges. Up to now, the country has experienced two waves (10). Recent data reported 21.128 confirmed cases and 387 deaths (9). As of the end of September 2021, in the country there were a total of 14,199 positive cases diagnosed since the beginning of the epidemic and 181 deaths (case-fatality at 1.27%; average age of deceased = 69.7 years, 69% of deaths ≥ 60 years) (11). WHO reported for the country as of August 31, 2020, the contamination of 122 (8.8%) health workers out of a total of 1,375 confirmed cases with COVID-19 (12). At the end of September 2021, the Central Plateau region had 116 confirmed cases of COVID-19, including one (1) death (11). The management of severe cases of COVID-19 in the country was carried out in the tertiary level hospitals of the capital Ouagadougou and the city of Bobo Dioulasso. The regional hospital of Ziniaré, the reference hospital of the Central Plateau region, located 35 km from the capital Ouagadougou, referred all severe cases requiring resuscitation to the university hospitals of the city of Ouagadougou.

**Figure 1:**
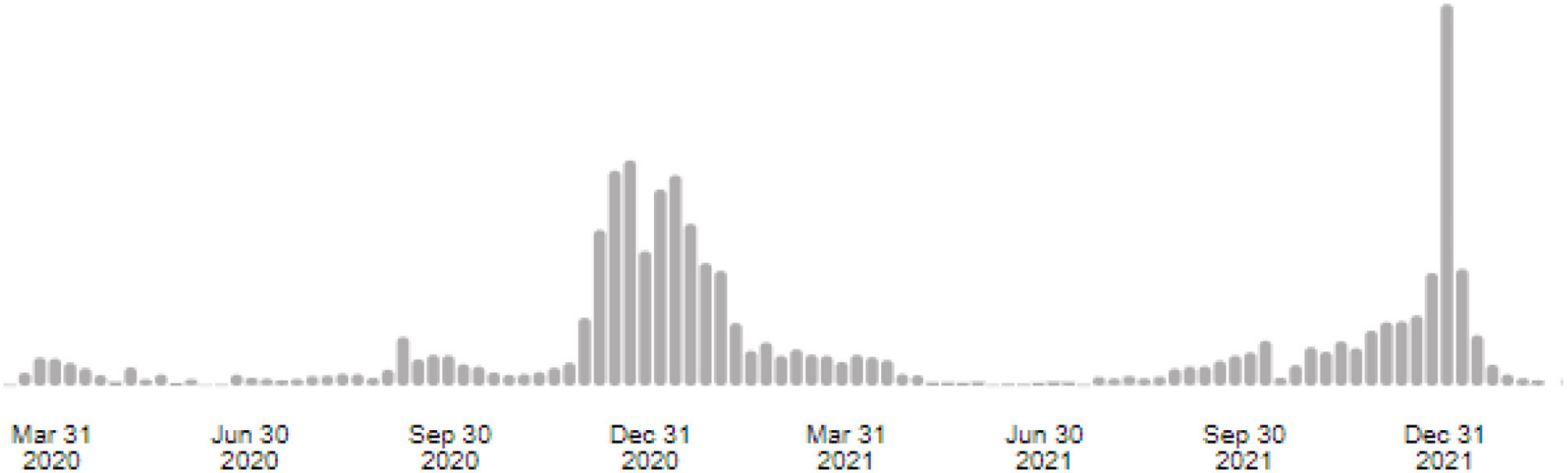
Progression of COVID-19 cases in Burkina Faso (13).

The Figure 1 shows the progression of COVID-19 cases in Burkina Faso, especially the peaks/waves and the period of study conduction (from September 20 to October 20, 2021) (13).

In the response to COVID-19, frontline health workers are inevitably at risk of SARS-CoV-2 infection (7, 14–16). They have a 3.4 times higher risk of contracting COVID-19, a higher risk of death compared to people living in the general community (17, 18). The fear of being infected is significant and related to the perceived risk of lethality. This fear generates stress, which affects all socio-professional categories independently of exposure to COVID-19 (5, 14, 19). Chaotic communication, in addition to supply problems (Polymerase Chain Reaction test, anti-COVID-19 drugs, personal protective equipment) was also a factor in increasing stress (4–6, 20). The reorganization of family life, isolation or social stigmatization in case of a positive test, the care of children who have left school, and the fear of contaminating family members, especially the elderly, are other sources of anxiety outside the hospital environment (1–6).

Studies of hospital staff in Europe, Asia and North America have found that caregivers involved in the care of COVID-19 positive patients have moderate to severe anxiety manifestations related to the infection itself, and to the fear of contamination of close contacts (7, 8, 14, 16, 19, 21–28).

To date, although the literature reports that health care workers, on the front line of the response, generally share the highest burden (1–6), few studies have assessed the impact of COVID-19 among hospital staff in Burkina Faso. This impact is usually underestimated in particular in developing countries (28). We provide information to answer the following question. What is the level of stress related to COVID-19 among health care workers in the Central Plateau health region? The objective of this study was to assess the extent of stress experienced by actors in the response to COVID-19 in the Plateau Central healthcare region of Burkina Faso.

## Materials and methods

### Type, period and setting of study

We conducted a cross-sectional study in the health districts and the regional hospital center of Plateau Central healthcare region of Burkina Faso from September 20 to October 20, 2021. The Plateau Central healthcare region has three health districts (Ziniaré, Boussé, and Zorgho) and one regional hospital. The population of the health region is estimated at 1,022,628 inhabitants. The Health Department staff during the study period was 1,355 (29). Recent cumulative data reported 187 confirmed cases of COVID-19 in the Plateau Central healthcare region (30).

Burkina Faso is a landlocked country in West Africa surrounded by six countries: Mali to the northwest, Niger to the northeast, Benin and Togo to the southeast, Ghana to the south and Côte d'Ivoire to the southwest. It has an area of 274,200 km^2^ and an estimated population of 21,509,443 in 2021 (31).

According to the World Health Organization, the health system is defined in three reference levels according to the technical platform (health care offer). The first level is local. It is the first contact level (nursing and medical care) and the first reference level (general medicine or medical specialty) and includes the sector and the health district, respectively. At this level, medical and nursing care is provided, respectively, in medical centers with a surgical unit (CMA), medical centers and health and social promotion centers (CSPS), dispensaries, and medical offices. At this first level of the health pyramid, community health centers are set up if access to health care and services is difficult. The second level is regional. It includes regional university or non-university hospital centers and polyclinics/clinics. This is the second level of reference. The third level is national. It includes national university hospitals and non-university hospitals that provide third-level care (32). This is the third level of reference. Among these neighboring countries, Burkina Faso is one of the first countries, along with Togo, to record confirmed cases of COVID-19 in 2020.

All personnel working at public healthcare centers (health care workers and administrative personnel) were eligible to complete the survey.

### Data collection

An anonymous questionnaire previously tested in the internal medicine department of the Yalgado Ouédraogo Teaching Hospital, one of the national referral hospitals in the city of Ouagadougou. It is a multipurpose department with two wards and a day hospital. About thirty hospital practitioners and students at the end of their medical studies responded to the questionnaire. After the questionnaire was sent to the managers of all the public healthcare centers in the health region.

Beforehand, a circular from the regional management of the central plateau, signed by the Regional Director of Health, announced the study to the practitioners in the region. Using the close-up strategy, the questionnaires were given to the different managers of the health structures concerned (chief doctors of the three health districts and director of the regional reference center). Under the supervision of the latter, the questionnaires were distributed to the health workers through the heads of department and unit managers and the head nurses. The completed forms were returned by the investigation team in reverse order by grouping the forms and forwarding them.

### Variables

The study outcome was the stress experienced by the workers assessed by the Perceived Stress Scale 10 (PSS-10) (33) and by a subjective stress rating scale. PSS-10 assesses the extent to which a person has perceived life as unpredictable, uncontrollable and overloaded during the past month. This scale consists of 10 questions. The questions are about the respondent's feelings and thoughts during the past month. Each question is rated from 0 to 4. By summing the responses to the ten questions, a score is calculated. This score was defined as low, moderate and high, respectively, when it was between 0 and 13, between 14 and 26 and between 27 and 40. The subjective stress rating scale had 10 points [1 (lower) to 10 (highest)]. The subjective stress was qualified as low, moderate and severe for 1 to 3, 4 to 6 and 7 to 10 points, respectively. The perceived stress and PSS-10 were assessed retrospectively for the most stressful period since the advent of COVID-19. On a 10-point scale, infectious risk was rated by health care staff. This risk was qualified as low, moderate and severe for 1 to 3, 4 to 6 and 7 to 10 points, respectively. Socio-demographic characteristics, psychiatric history, medical history, knowledge and perceptions of COVID-19 and perceived stress were also collected.

### Data analysis

The dependent variable was perceived stress as measured by the PSS10 coded in two modalities (low or moderate/high). For descriptive analysis, categorical variables were presented by their frequency and percentage; quantitative variables by their mean and standard deviation. Factors associated with high stress according to PSS10 (>27) during the period in which the stress was most felt were analyzed using logistic regression (34). Among the various measures of stress, the Perceived Stress Scale (PSS) is widely used and has been the subject of numerous studies. These studies have demonstrated its satisfactory psychometric qualities (35–42). Relevant independent variables were selected for univariate and multivariate analysis. Factors associated in the univariate analysis at the 20% threshold were selected for the multivariate analysis. The final model was obtained by the stepwise manual descending strategy. The significance level in the final model was 5%. All these analyses were performed with STATA version 15.1.

### Ethical considerations

For this study, approval and authorization from the Central Plateau regional health directorate and all health district officials (chief physicians) and the regional hospital center (general manager) were obtained before the survey was conducted. Attached is the survey authorization from the Central Plateau Regional Health Department.

Written consent was obtained from the participants before any response to the self-administered questionnaire.

## Results

Overall, 800 questionnaires were sent to health care workers and 272 subjects (34%) responded to the questionnaire. The mean age of the study participants was 37.11 years [Standard Deviation (SD): 6.33 years] with extremes of 20 and 56 years. The majority of subjects was men (160, 58.82%). The average seniority of the staff was 8.97 years (standard deviation: 6.24 years) with extremes of one and 32 years. Seven (2.57%) workers had a psychiatric history. Table 1 reports the socio-demographic characteristics and medical history of the study participants.

**Table 1:** Socio-demographic characteristics and medical history of public health center staff in the Plateau Central healthcare region (Burkina Faso).

	**Frequency**	**Percentage (%)**
**Healthcare decision area**		
Regional Hospital Center of Ziniaré	82	30.15
Healthcare District of Boussé	56	20.59
Healthcare District of Ziniaré	64	23.53
Healthcare District of Zorgho	70	25.74
**Level of the healthcare pyramid**		
Health and Social Promotion Center	159	58.46
Medical Center with Surgical Branch/Medical Center	33	12.50
Regional Hospital Center	79	29.04
**Type of personnel**		
Non-caregiver	20	7.35
Medical staff	31	11.40
Paramedical staff	221	81.25
**Age (years)**		
<35	90	33.09
[35–45]	149	54.78
[45–55]	31	11.40
≥55	2	0.74
**Gender**		
Male	160	58.82
Female	112	41.18
**Seniority in the position (years)**		
<5	73	27.34
[5–10]	85	31.84
≥10	109	40.82
**Social status**		
Lives alone	36	13.33
Lives in a family with children	217	80.37
Living with people at risk (diabetic, hypertensive patients)	17	6.30
**Level of education**		
Middle school	160	59.26
College	110	40.74
**Psychiatric history**		
Yes	7	2.57
No	265	97.43
**Other types of medical history**		
Diabetes	6	2.21
Hypertension	19	6.99
Tuberculosis	3	1.1
Hepatitis	1	0.37
Other	13	4.78

Two hundred and twenty (80.88%) participants were aware of COVID-19 through the media. The infectious risk of COVID was reported by 270 (99.26%) subjects. In this context, 64 (23.53%) respondents had been tested for COVID-19 at least once. Of these, 13 (20.31%) had reported testing positive. Among respondents, 58 (21.40%) reported having helped manage a COVID-19 positive patient. Exposure to possible COVID-19 contamination by patients was found in 114 (41.91%) of them. Table 2 presents the perceptions of the study participants about COVID-19.

**Table 2:** Public health center staff perception and COVID-19 experience in the Plateau Central healthcare area (Burkina Faso).

	**Frequency**	**Percentage**
**Is COVID-19 an infectious risk?**		
I do not know	1	0.37
No	1	0.37
Yes	270	99.26
**Infectious risk gradation scale [points]**		
No risk and low risk [1–3 points]	27	9.96
Moderate risk [4–6 points]	44	16.24
High risk [7–10 points]	200	73.80
**Have been tested for COVID-19**		
I do not know	4	1.47
No	204	75.00
Yes	64	23.53
**Result of the COVID-19 test**		
I do not know	7	10.94
Negative	44	68.75
Positive	13	20.31
**Management of a COVID-19 patient**		
I do not know	2	0.74
No	211	77.86
Yes	58	21.40
**A relative tested COVID-19 positive**		
I do not know	13	4.78
No	193	70.96
Yes	66	24.26
**A colleague tested COVID-19 positive**		
I do not know	3	1.10
No	186	68.38
Yes	83	30.51
**Exposure to a risk of contamination by COVID-19**		
One of your relatives (family, friends)	74	27.21
One of your colleagues	89	32.72
One of your patients	114	41.91
No	81	29.78
I do not know	8	2.94
**Sources of information on COVID-19**		
Scientific	84	30.88
Governmental	142	52.21
Hospital	123	44.85
Media	220	80.88
Surroundings	55	20.22

The mean PSS-10 score was 29.30 (Standard deviation: 6.22 points). Among respondents, 185 (68.01%) had a high level of stress according to the PSS-10. The subjective stress rating scale found high levels of stress in 186 (68.63%) respondents. The period of greatest stress for 206 (78.33%) respondents was during the first wave of the pandemic in Burkina Faso (March to June 2020). The sources of stress were the fear of seeing a large number of COVID-19 positive patients in hospitals for 164 (60.29%), the risk to be exposed to contamination (190, 69.85%) and to be the source of contamination (213, 78.31%). Table 3 presents the symptoms, sources and stressors related to COVID-19.

**Table 3:** COVID-19-related stress experienced by public health workers in the Plateau Central healthcare region (Burkina Faso) on COVID-19.

	**Frequency**	**Percentage**
**Stress felt since the COVID-19**		
I do not know	2	0.74
No	16	5.88
Yes	254	93.38
**Stress related to the COVID-19**		
I do not know	10	3.92
No	22	8.63
Yes	223	87.45
**Subjective grading of stress experienced during the first wave [points]**		
Low [1–3 points]	25	9.23
Moderate [4–6 points]	60	22.14
High [7–10 points]	186	68.63
**Gradation of stress experienced according to the PSS-10 during the first wave [points]**		
Low [0–14 points]	1	0.37
Moderate [14–27 points]	86	31.62
High [27–40 points]	185	68.01
**Period of highest stress level**		
March 2020 to June 2020	206	78.33
July 2020 to October 2020	17	6.46
November 2020 to January 2021	24	9.13
January 2021 to present day	16	6.08
**Stress factors or sources of stress in COVID-19**		
Fear of COVID-19 positive patients hospitalized in the health center	164	60.29
Risk of being contaminated	190	69.85
Risk of infecting family and friends	213	78.31
Risk of death from COVID-19	99	36.40
Severity of COVID-19	72	26.47
Insufficient or conflicting information on COVID-19	85	31.25
Lack of personal protective equipment	94	34.56
Lack of PCR tests at the beginning of the epidemic	79	29.04

In univariate analysis, the level of the health pyramid, getting information from scientific sources, hospital sources, the fear of seeing severe cases of COVID-19 positive patients in hospitals, the risk of being a source of contamination for one's close relatives, and have been tested for COVID-19 were the variables statistically associated with high stress among subjects working in the health sector during the first wave of COVID-19 in Burkina Faso. The positivity of a close relative to COVID-19, as well as that of a colleague and the risk of being exposed to contamination through close relatives or friends, were also variables statistically associated with high stress among health workers in the Plateau Central healthcare area during the first wave of COVID-19 in Burkina Faso.

In multivariate analysis, the level of health center in the health pyramid [adjusted Odds Ratio (aOR): 2,29; 95% Confidence Interval (95%CI): 1,19–4,41], the hospital as a source of information [aOR = 1.17; 95% CI (1.01–3.04)], and the fear of seeing severe cases of COVID-19 hospitalized in the health center (aOR: 1,8; 95% CI: 1,06–3,07) were the factors independently associated with high stress according to PSS-10 among health workers in the Plateau Central healthcare region during the first wave of COVID-19 in Burkina Faso (Table 4).

**Table 4:** Factors associated with stress as measured by the PSS-10 among public health workers in the Plateau Central healthcare region (Burkina Faso) during COVID-19 1^st^ wave.

	**Stress experienced during the first wave according to PSS10 score**					
	**Univariate**			**Multivariate**		
	**OR**	**CI 95%**	***p-*** **value**	**aOR**	**CI 95%**	***p*** **-value**
**Gender**						
Male	1	-	-			
Female	0.85	[0.51–1.43]	0.56			
Level of the healthcare pyramid			<0.01			
Health centers at the district level^*^	1	-	-			
Regional hospital center	2.53	[1.34–4.78]	<0.01	2.29	[1.19–4.41]	0.01
Type of personnel			0.10			
Non-caregiver	1	-	-			
Medical staff	1.73	[0.43–6.97]	0.43			
Paramedical staff	0.62	[0.21–1.78]	0.37			
Age (in years)			0.60			
<35	1	-	-			
[35–45]	1.29	[0.73–2.26]	0.36			
[45–55]	0.83	[0.35–1.93]	0.66			
≥ 55	0.52	[0.03–8.68]	0.65			
Seniority in the position (years)			0.76			
<5	1	-	-			
[5–10]	0.77	0.39–1.49	0.47			
≥10	0.85	0.44–1.61	0.63			
Social status			0.90			
Lives alone	1	-	-			
Lives in a family with children	1.11	0.52–2.37	0.76			
Living with people at risk s(Diabetic, hypertensive, patients)	0.91	0.27–3.08	0.88			
**Level of education**						
Middle school	1	-	-			
College	1.43	0.84–2.44	0.18			
**Sources of information's about COVID-19**						
Scientific (ref: no)	2.33	[1.27–4.29]	<0.01			
Governmental (ref: no)	1.54	[0,92–2,58]	0.09			
Hospital (ref: no)	2.02	[1.18–3.43]	<0.01	1.17	[1.01–3.04]	0.04
Media (ref: no)	1.42	[0.76–2.67]	0.26			
Surroundings (ref: no)	1.48	[0.76–2.89]	0.24			
**Infectious risk gradation scale [points]**						
No risk and low risk [0–3 points]	1	-	-			
Moderate risk [4–6 points]	2	[0.69–5.72]	0.19			
High risk [7–10 points]	1.16	[0.50–2.68]	0.71			
**Have been tested for COVID-19 (ref: no)**						
Yes	3.13	[1.50–6.52]	<0.01			
**Positivity of a relative to COVID-19 screening test (ref: no)**						
Yes	2.39	[1.19–4.78]	0.01			
**Positivity of a colleague at COVID-19 screening test (ref: no)**						
Yes	2.52	[1.35–4.70]	<0.01			
**Management of a COVID-19 patient (ref: no)**						
Yes	1.79	[0.90–3.53]	0.09			
**Subjective grading of stress experienced during the first wave [points]**						
Low [0–3 points]	1	-	-			
Moderate [4–6 points]	1.38	[0.54–3.54]	0.49			
High [7–10 points]	2.44	[1.04–5.70]	0.03			
**Stress factors or sources of stress in COVID-19**						
Fear of COVID-19 positive patients hospitalized in the health center	1.80	[1.07–3.02]	0.02	1,80	[1.06–3.07]	0.02
Risk of being contaminated by family and friends (ref: no)	1.45	[0.84–2.50]	0.17			
Risk of infecting family and friends ref: no)	1.96	[1.08–3.55]	0.02			
Risk of death from COVID-19 (ref: no)	0.90	[0.53–1.53]	0.71			
Severity of COVID-19 (ref: no)	1.58	[0.86–2.90]	0.14			
Insufficient or conflicting information on COVID-19 (ref: no)	0.74	[0.43–1.27]	0.28			
Lack of personal protective equipment (ref: no)	1.36	[0.78–2.35]	0.26			
Lack of PCR tests at the beginning of the epidemic (ref: no)	1.56	[0.87–2.82]	0.13	2.25	[1.15–4.35]	0.01

* Health center at a district level were Health and Social Promotion Center, Medical Center with Surgical Branch and Medical Center. The final level of referral for health care in the region is the regional hospital.

## Discussion

In light of the paucity of studies that have assessed the impact of COVID-19 among hospital workers in Africa, this study from Burkina Faso could have a significant added value to the literature. It is helpful to highlight the stress experienced by actors in response to COVID-19 using the Perceived Stress Scale 10 (PSS-10) in a developing country.

The majority of health care center staff in the Plateau Central healthcare area included in this study were unanimous about the potential infectious risk of COVID-19 in health care settings.

A high level of stress, both subjective and with the PSS-10 score (68%), was also experienced during the first wave of the pandemic in Burkina Faso. The COVID-19 pandemic was a source of stress and had an effect on staff in both their social and professional spheres. However, a small proportion of workers reported having been tested for COVID-19. The level of the health center in the healthcare pyramid, hospital (health center) as a source of information, fear of seeing hospitalized severe case of COVID-19 in the health center were independently associated with high stress among health care center staff in the Plateau Central healthcare region during the first wave of COVID-19 in Burkina Faso.

To break the chain of transmission of SARS-CoV-2 in Burkina Faso, decisions were implemented by health authorities, including barrier and social distancing measures, as well as containment (20). These decisions followed the declaration of the epidemic in Burkina Faso, corresponding to the start and first wave from March to June 2020. It was during this period that staff reported experiencing the highest level of stress (78.33%).

During the first wave of the COVID-19 pandemic, seven out of ten experienced high stress level in Burkina Faso. This pandemic was unique in that it was sudden, uncertain and unknown (1–6). The African continent did not experience the impact predicted in the beginning of the COVID-19 pandemic. However, the health system has still experienced shocks (43, 44). In Burkina Faso, the response to the COVID-19 pandemic took place in a context marked by the inadequacy of the health system in terms of infrastructure and logistics. The humanitarian needs due to insecurity were also making the settings of the COVID-19 response harder (20). The management of the COVID-19 pandemic in the country was also marked by controversies both in the community and among health care workers (20). Indeed, at the early stage of the pandemic the first reported case of death caused a national outcry and was the cause of media chaos and increased stress at the beginning of the pandemic (44). The first wave was associated with panic, anxiety, fear of infection and fear of death. Our study confirms this as a source of stress for workers. Indeed, in multivariate analysis, government information relayed in hospitals would significantly increase the risk of high stress among health care workers in the Plateau Central healthcare area [aOR = 1.17; 95% CI (1.01–3.04)].

In this anxiety-provoking environment, only one in five health care center staff had been tested for COVID-19. Of these, 24% reported having tested positive for COVID-19. This low proportion of screening could be explained by the fact that at the beginning of the pandemic, only suspected cases or contacts of confirmed or probable cases were screened. Gradually, frontline workers in the pandemic response and people at risk were targeted for screening. In our study, working in the regional hospital (the reference health center of the region) significantly increased the risk of stress in health workers (aOR: 2.29; CI95% [1.19–4.41]). This association could be explained by the fear of testing positive in a context of organizational inadequacy and lack of treatment. Indeed, during the first wave, the contact subjects were quarantined and forbidden to visit their relatives (20), which could aggravate the stress felt. The management of positive cases was only hospital-based and severe cases required oxygen therapy which was often unavailable. Data from the literature show that front-line health workers are inevitably exposed to the risks of SARS-CoV-2 infection (15). They have a 3.4 times higher risk of contracting COVID-19 compared to people living in the general community (17). The WHO reports that infection of health workers accounts for 5% of positive cases in Africa (45). This incidence in African countries is highly variable, but is increasing (46). In Tunisia, the rate of positivity among front-line workers was 14.4% (47). In Burkina Faso, it was 8.8% (12).

In addition, a small proportion (21.40%) of the participants in this study was involve in the management of a patient tested positive for COVID-19. This low proportion can be explained by the fact that at the beginning of the pandemic in Burkina Faso, the management of COVID-19 cases was done in university hospitals, then in regional hospitals before being decentralized to health and social promotion centers and home-based care. The late decentralization of the management of COVID-19 positive cases to the regional and local levels of the health system may have contributed to increased stress among workers by creating a myth about the coronavirus disease among these staff who were not initially involved in the response but were overwhelmed with information about the measures or precautions to be taken in the services and the restrictions observed in society (20).

The pandemic of coronavirus (COVID-19) has a social and psychological impact on the mental health of health workers worldwide (1–8). However, few studies have been conducted in developing countries. Methodological differences also make it difficult to compare with the results of our study.

Nevertheless, data from the literature reveal a significant increase in stress-related symptoms (Anxiety, Depressive Symptoms, Insomnia, Burnout, and Functional Impairment) among health care workers during the COVID-19 pandemic. This stress is increased due to their pre-existing limited access and resources, imposed by the colonial system, and their management of the new coronavirus, in certain vulnerable subgroups (health care workers) who are likely to require psychological interventions). Indeed significant associations were observed between attitude toward interprofessional teamwork, gender, marital status, occupation, work experience, current work location (clinics), spiritual influences, perceived competence, difficulties in daily life, income level, confidence in individual instincts, level of control over aspects of resilience, provision of COVID-19 patient care, history of COVID-19 testing, history of COVID-19 testing or infection, and availability of mental health support in the workplace (7, 8, 14, 16, 19, 24–28, 48).

Interpretation of our results must take into account certain limitations. Our data were collected only in the Plateau Central healthcare area. Our sample is therefore not representative of all health workers in Burkina Faso. Above one third of the questionnaires sent out were returned. Participation in the study was voluntary. These expose to a potential selection bias that could underestimate or overestimate the proportion of workers with high levels of stress. We assessed the stress reported by the workers in a subjective and objective manner by collecting their statements. This method of data collection may expose potential information bias. We believe, however, that this bias was minimized by the fact that we used completely anonymous validated self-questionnaires. Despite these limitations, our study is original and is the first to estimate the level of stress in a subjective and objective manner among health workers in Burkina Faso. These data will fill the knowledge gap in the field and allow the health system to better prepare for the response to future epidemics.

## Conclusion

The COVID-19 pandemic caused high stress among health workers in Burkina Faso, mainly during the first wave. Psychological support for health center workers in the response to future epidemics would improve their mental health.

## Data availability statement

The raw data supporting the conclusions of this article will be made available by the authors, without undue reservation.

## Ethics statement

The studies involving human participants were reviewed and approved by Regional Healthcare Directorate of the Plateau Central healthcare region, Burkina Faso. Written informed consent for participation was not required for this study in accordance with the national legislation and the institutional requirements.

## Author contributions

ST, DD, and BP conceived the study, participated in its design, and helped to draft the manuscript. YL, WK, W-L-SO, DKam, WS, DKab, AO, SS, MZ, BO, and OG helped in the coordination. All authors read and approved the final manuscript.

## References

[1] Pearman A Hughes ML Smith EL Neupert SD. Mental health challenges of united states healthcare professionals during COVID-19. Front Psychol. (2020) 11:2065. 10.3389/fpsyg.2020.02065 32903586 PMC7438566

[2] Chen Q Liang M Li Y Guo J Fei D Wang L. Mental health care for medical staff in China during the COVID-19 outbreak. Lancet Psychiatry. (2020) 7:e15-6. 10.1016/S2215-0366(20)30078-X 32085839 PMC7129426

[3] Martín J Padierna Á Villanueva A Quintana JM. Evaluation of the mental health of health professionals in the COVID-19 era. what mental health conditions are our health care workers facing in the new wave of coronavirus? Int J Clin Pract. (2021) 75:e14607. 10.1111/ijcp.14607 34231287 PMC8420292

[4] Mboua CP Keubo FRN Fouaka SGN. Anxiété et dépression associées à la prise en charge de la COVID-19 chez les personnels de santé au Cameroun. É*vol Psychiatr*. (2021) 86:131–9. 10.1016/j.evopsy.2020.11.002 33318714 PMC7724313

[5] Georger F Dos Santos E Gazagne L Berdagué P Saib A Nahon S. COV IMPACT: analyse des différents facteurs de stress du personnel hospitalier dans 2 centres hospitaliers en France lors de la pandémie COVID-19. Ann Cardiol Angeiol. (2020) 69:227–32. 10.1016/j.ancard.2020.09.005 33059875 PMC7510417

[6] El-Hage W Hingray C Lemogne C Yrondi A Brunault P Bienvenu T. Les professionnels de santé face à la pandémie de la maladie à coronavirus (COVID-19): quels risques pour leur santé mentale ? Encephale. (2020) 46:S73-80. 10.1016/j.encep.2020.04.008 32370984 PMC7174182

[7] Elsayed MEG El-Abasiri RA Marzo RR Dardeer KT Kamal MA Abdelaziz H. Mental health, risk perception, and coping strategies among healthcare workers in Egypt during the COVID-19 pandemic. PLoS ONE. (2023) 18:e0282264. 10.1371/journal.pone.0282264 36848375 PMC9970061

[8] Hwaij RA Ghrayeb F Marzo RR AlRifai A. Palestinian healthcare workers mental health challenges during COVID-19 pandemic: a cross-sectional study. Med. Res. Arch. (2022) 10:1–9. 10.18103/mra.v10i10.3125

[9] Dong E Ratcliff J Goyea TD Katz A Lau R Ng TK. The johns hopkins university center for systems science and engineering COVID-19 dashboard: data collection process, challenges faced, and lessons learned. Lancet Infect Dis. (2022) 22:e370–6. 10.1016/S1473-3099(22)00434-0 36057267 PMC9432867

[10] Somda SMA Ouedraogo B Pare CB Kouanda S. Estimation of the serial interval and the effective reproductive number of COVID-19 outbreak using contact data in Burkina Faso, a Sub-Saharan African Country. Comput Math Methods Med. (2022) 2022:8239915. 10.1155/2022/8239915 36199779 PMC9527438

[11] Centre des Operations de Reponse aux Urgences Sanitaires (CORUS). Rapport de situation sur l'épidémie de la maladie à Coronavirus (COVID-19) au Burkina Faso. Centre des Operations de Reponse aux Urgences Sanitaires (CORUS) (2021).

[12] Organisation mondiale de la santé. Organisation mondiale de la santé-COVID-19 au Burkina Faso : les 6 premiers mois de l'OMS aux coté du pays dans la riposte. Bulletin spécial 6 mois, Mars-Aout 2020. Organisation mondiale de la santé (2020).

[13] Burkina Faso. WHO Coronavirus Disease (COVID-19) Dashboard With Vaccination Data. Available online at: https://covid19.who.int (accessed April 23, 2023).

[14] Yassin A Al-Mistarehi AH Soudah O Karasneh R Al-Azzam S Qarqash AA. Trends of prevalence estimates and risk factors of depressive symptoms among healthcare workers over one year of the COVID-19 pandemic. Clin Pract Epidemiology Ment Health. (2022) 18:1–16. 10.2174/17450179-v18-e2206160 PMC10158079 37274865

[15] Folgueira MD Muñoz-Ruipérez C Alonso-López MÁ Delgado R. SARS-CoV-2 infection in health care workers in a large public hospital in Madrid, Spain, during March 2020. Infect Dis. (2020) 106:357–63. 10.1101/2020.04.07.20055723

[16] Yassin A Al-Mistarehi AH El-Salem K Karasneh RA Al-Azzam S Qarqash AA. Prevalence estimates and risk factors of anxiety among healthcare workers in jordan over one year of the COVID-19 pandemic: a cross-sectional study. Int J Environ Res Public Health. (2022) 19:2615. 10.3390/ijerph19052615 35270333 PMC8909996

[17] Nguyen LH Drew DA Graham MS Joshi AD Guo CG Ma W. Risk of COVID-19 among front-line health-care workers and the general community: a prospective cohort study. Lancet Public Health. (2020) 5:e475-83. 10.1016/S2468-2667(20)30164-X 32745512 PMC7491202

[18] Gaber DM Ahmed MM Sayed AM Elkholy YS Sarhan MD. Perception of COVID-19-related stigma and associated psychological challenges among healthcare workers at Cairo University hospitals. J Int Med Res 1 janv. (2023) 51:3000605221148833. 10.1177/03000605221148833 36650917 PMC9869217

[19] Yusefi AR Faryabi R Bordbar S Daneshi S Nikmanesh P. Job burnout status and its relationship with resilience level of healthcare workers during Covid-19 pandemic: a case of Southern Iran. Iranian J Health Science. (2021) 9:1-11. 10.18502/jhs.v9i3.7305

[20] Kobiané JF Soura BA Sié A Ouili I Kabore I Guissou S. Les inégalités au Burkina Faso à l'aune de la pandémie de la COVID-19 : quelques réflexions prospectives: In: Les inégalités au Burkina Faso à l'aune de la pandémie de la COVID-19: quelques réflexions prospectives. Agence française de développement. (2020) 1–72. 10.3917/afd.kobia.2020.01.000118052372

[21] Koh D Lim MK Chia SE Ko SM Qian F Ng V. Risk perception and impact of severe acute respiratory syndrome (SARS) on work and personal lives of healthcare workers in Singapore: what can we learn? Med Care. (2005) 43:676–82. 10.1097/01.mlr.0000167181.36730.cc 15970782

[22] Goulia P Mantas C Dimitroula D Mantis D Hyphantis T. General hospital staff worries, perceived sufficiency of information and associated psychological distress during the A/H1N1 influenza pandemic. BMC Infect Dis déc. (2010) 10:322. 10.1186/1471-2334-10-322 21062471 PMC2990753

[23] Matsuishi K Kawazoe A Imai H Ito A Mouri K Kitamura N. Psychological impact of the pandemic (H1N1) 2009 on general hospital workers in Kobe: pandemic in Kobe. Psychiatry Clin Neurosci. (2012) 66:353–60. 10.1111/j.1440-1819.2012.02336.x 22624741

[24] Htay MNN Marzo RR AlRifai A Kamberi F El-Abasiri RA Nyamache JM. Immediate impact of COVID-19 on mental health and its associated factors among healthcare workers: a global perspective across 31 countries. J Glob Health. (2020) 10:020381. 10.7189/jogh.10.020381 33214890 PMC7649521

[25] Marzo RR Khaled Y ElSherif M Abdullah MSAMB Zhu Thew H Chong C. Burnout, resilience and the quality of life among Malaysian healthcare workers during the COVID-19 pandemic. Front Public Health. (2022) 10:1021497. 10.3389/fpubh.2022.1021497 36530707 PMC9747946

[26] Rillera Marzo R Elsherif M Bin Abullah MSAM Thew HZ Chong C Yih Soh S. Demographic and work-related factors associated with burnout, resilience, and quality of life among healthcare workers during the COVID-19 pandemic: a cross sectional study from Malaysia. Front Public Health. (2022) 10:1021495. 10.3389/fpubh.2022.1021495 36589987 PMC9800419

[27] Yassin A Al-Mistarehi AH Qarqash AA Soudah O Karasneh RA Al-Azzam S. Trends in insomnia, burnout, and functional impairment among health care providers over the first year of the COVID-19 pandemic. Clin Pract Epidemiol Ment Health. (2022) 18:1–20. 10.2174/17450179-v18-e2206200 PMC10156054 37274859

[28] Kamberi F Sinaj E Jaho J Subashi B Sinanaj G Jaupaj K. Impact of COVID-19 pandemic on mental health, risk perception and coping strategies among health care workers in Albania-evidence that needs attention. Clin Epidemiol Glob Health. (2021) 12:100824. 10.1016/j.cegh.2021.100824 34751254 PMC8567021

[29] Ministère de la santé-Burkina Faso. Ministère de la santé-Burkina Faso. Annuaire statistique 2021. Direction générale des études et des statistiques sectorielles. Direction générale des études et des statistiques sectorielles (2022).

[30] Ministère de la santé-Burkina Faso. Ministère de la santé-Burkina Faso Rapport de situation sur l'épidémie de la maladie à Coronavirus (COVID-19) dans la région sanitaire du Plateau Central Directions Régionale de la santé (2022).

[31] Institut National de la Statistique et de la Demographie. Cinquième Recensement Général de la Population et de l'Habitation du Burkina Faso INSD Burkina Faso (2022).

[32] Ministère de la Santé, Burkina Faso. Profil sanitaire complet du Burkina Faso : Système de santé du Burkina Faso. (2017). Available online at: https://www.afro.who.int/sites/default/files/2018-08/Profil%20sanitaire%20du%20Burkina%20%202.pdf

[33] Bellinghausen L Collange J Botella M Emery JL Albert E. Factorial validation of the French scale for perceived stress in the workplace. Sante Publique. (2009) 21:365–73. 10.3917/spub.094.0365 20101815

[34] Cohen S Kamarck T Mermelstein R. A global measure of perceived stress. J Health Soc Behav. (1983) 24:385-96. 10.2307/2136404 6668417

[35] Schneider EE Schönfelder S Domke-Wolf M Wessa M. Measuring stress in clinical and nonclinical subjects using a German adaptation of the Perceived Stress Scale. Int J Clin Health Psychol. (2020) 20:173–81. 10.1016/j.ijchp.2020.03.004 32550857 PMC7296237

[36] Bastianon CD Klein EM Tibubos AN Brähler E Beutel ME Petrowski K. Perceived Stress Scale (PSS-10) psychometric properties in migrants and native Germans. BMC Psychiatry. (2020) 20:450. 10.1186/s12888-020-02851-2 32917170 PMC7488568

[37] Anwer S Manzar MD Alghadir AH Salahuddin M Abdul Hameed U. Psychometric analysis of the perceived stress scale among healthy university students. Neuropsychiatr Dis Treat. (2020) 16:2389–96. 10.2147/NDT.S268582 33116538 PMC7585521

[38] Meng R Li J Wang Z Zhang D Liu B Luo Y. The Chinese version of the Perceived Stress Questionnaire: development and validation amongst medical students and workers. Health Qual Life Outcomes. (2020) 18:70. 10.1186/s12955-020-01307-1 32169070 PMC7071673

[39] The Thai version of the PSS-10: An Investigation of its psychometric properties. *SpringerLink*. Available online at: https://link.springer.com/article/10.1186/1751-0759-4-6 (accessed April 24, 2023).

[40] Chen JY Chin WY Tiwari A Wong J Wong ICK Worsley A. Validation of the perceived stress scale (PSS-10) in medical and health sciences students in Hong Kong. TAPS. (2021) 6:31-7. 10.29060/TAPS.2021-6-2/OA2328

[41] Soria-Reyes LM Cerezo MV Alarcón R Blanca MJ. Psychometric properties of the perceived stress scale (pss-10) with breast cancer patients. Stress Health. (2023) 39:115–24. 10.1002/smi.3170 35657280 PMC10084090

[42] Flores-Torres MH Tran A Familiar I López-Ridaura R Ortiz-Panozo E. Perceived stress scale, a tool to explore psychological stress in Mexican women. Salud Publica Mex. (2021) 64:49–56. 10.21149/12499 35438916

[43] Amu H Dowou RK Saah FI Efunwole JA Bain LE Tarkang EE. COVID-19 and health systems functioning in sub-saharan africa using the “who building blocks”: the challenges and responses. Front Public Health. (2022) 10:856397. 10.3389/fpubh.2022.856397 35444973 PMC9013894

[44] Tessema GA Kinfu Y Dachew BA Tesema AG Assefa Y Alene KA. The COVID-19 pandemic and healthcare systems in Africa: a scoping review of preparedness, impact and response. BMJ Glob Health déc. (2021) 6:e007179. 10.1136/bmjgh-2021-007179 34853031 PMC8637314

[45] Coronavirus en Afrique. Coronavirus en Afrique : Quels sont les pays impactés? (2020).

[46] Nkengasong JN Mankoula W. Looming threat of COVID-19 infection in Africa: act collectively, and fast. Lancet. (2020) 395:841–2. 10.1016/S0140-6736(20)30464-5 32113508 PMC7124371

[47] Mrazguia C Aloui H Fenina E Boujnah A Azzez S Hammami A. L'infection par le COVID-19 chez le personnel de santé à l'hôpital Régional de Nabeul : épidémiologie et circonstances de transmission. PAMJ-OH. (2021) 4. 10.11604/pamj-oh.2021.4.11.27891

[48] Htay MNN Marzo RR Bahari R AlRifai A Kamberi F El-Abasiri RA. How healthcare workers are coping with mental health challenges during COVID-19 pandemic? - a cross-sectional multi-countries study. Clin Epidemiol Glob Health. (2021) 11:100759. 10.1016/j.cegh.2021.100759 33977169 PMC8103713

